# Do medical students feel career ready after their psychiatry clinical rotation?

**DOI:** 10.4102/sajpsychiatry.v25i0.1397

**Published:** 2019-11-29

**Authors:** Kim Ives, Piet J. Becker, Gian Lippi, Christina Krüger

**Affiliations:** 1 Department of Psychiatry, Faculty of Health Sciences, University of Pretoria, Pretoria, South Africa; 2 Research Office, Faculty of Health Sciences, University of Pretoria, Pretoria, South Africa

**Keywords:** medical education, psychiatry, undergraduate training, medical students, career preparedness, mental illness

## Abstract

**Background:**

In the South African healthcare system, mentally ill patients first come into contact with primary care physicians who then refer these patients for specialised care if needed. Medical students therefore need to acquire the knowledge, skills and confidence to treat mentally ill patients.

**Aim:**

To evaluate the perceptions of medical students regarding their career readiness as doctors after their clinical rotation in psychiatry.

**Setting:**

The University of Pretoria, South Africa.

**Methods:**

Data were collected retrospectively from questionnaires completed by final year medical students from 2011 to 2015. These data were analysed overall and by year using Chi-square tests and regression analyses (*N* = 770).

**Results:**

Overall, 93.10% of medical students felt adequately prepared for their role as medical practitioners after their clinical rotation in psychiatry. The proportion of medical students exposed to post-traumatic stress disorder (*p* = 0.012), obsessive-compulsive disorder (*p* = 0.006) and alcohol-use disorder (*p* = 0.046) was found to vary significantly by year. Exposure to any one psychiatric condition did not influence perceptions of career preparedness. Students perceived themselves to be career ready if they had sufficient exposure to mentally ill patients, knowledge about prescribing appropriate psychiatric medication and especially psychiatric interviewing skills.

**Conclusion:**

Students who completed practical and clinical training in psychiatry perceived themselves to be career ready.

## Introduction

In addition to objective exam results, the overall feeling of being career ready is important for transitioning successfully from being a student to becoming a qualified doctor. Exam results alone do not correlate with a student’s confidence or feelings of preparedness,^1^ especially for medical graduates who often feel ill-prepared for the demands of their new job.^2^ Most people starting a new job will probably feel unprepared to some extent. This is especially important in the healthcare sector, where inexperience and insecurity can lead to mistakes that affect patients’ health.^1^

In 2003, Jungbauer et al. reported that only about one third of graduates of seven German universities felt well or very well prepared for being a doctor after finishing medical school, and many graduates criticised the lack of practical relevance of the curriculum with regard to their upcoming career.^3^ Oschsmann et al. evaluated if specific medical competencies influenced the overall feeling of preparedness for caring for patients.^1^ They found that approximately 66% of participants did not feel well prepared for their job after finishing medical school.

### Student training in psychiatry

Medical education has distinctive features, and undergraduate medical curricula differ from other higher education curricula in many respects. For example, clinical teaching and problem-based learning are prominent features of medical education.^4^ Students whose undergraduate training included structured mentoring and career guidance reported stronger feelings of career readiness. Similarly, face-to-face clinical teaching is highly valued by students and should be prioritised during psychiatry rotations to build positive attitudes towards psychiatry.^5^

Work readiness may be encouraged if medical students have the opportunity to ask for advice from fellow students, junior doctors and senior doctors.^2^ Students also benefit from early, structured, work-based experiential learning, as evidenced by students performing internships in small rural medical schools in Australia.^6^ A longitudinal study conducted at a Polish medical school indicated that final year medical students were concerned about being taught ‘too little practice and too much theory’ and not having ‘adequate skills for future work’.^7^

Course evaluations by medical students are widely used to evaluate and improve teaching.^8^ Most medical schools ask students to rate the quality of their learning experiences, and then they use this feedback in efforts to improve curricula and courses.^9^ Many investigators have stated that student ratings are the most valid and practical source of data on teaching and course effectiveness.^4^ Self-perceived career readiness is linked to career crystallisation or realisation and is an important goal of medical education. Self-perceived career readiness is built through a process of self-reflection and critical thinking.^10^ Thus, medical students completing their courses need to be able to evaluate what they have been taught. In a study, exploring medical students’ views on effective teaching methods in psychiatry, it becomes clear that medical students are able to critique teaching methods in psychiatry.^11^

### The role of primary care physicians in the treatment of mentally ill patients

In South Africa, medical students complete their clinical rotations in their 5th and 6th years, upon which they qualify as health practitioners. Newly qualified medical doctors then have to complete 2 years of internship as junior doctors, providing healthcare to patients in the public sector. Many of these newly qualified doctors will come into contact with patients displaying psychiatric symptoms.

Yadav et al. note that the internship period is perceived as the most stressful, challenging and important period of a doctor’s working life.^12^ Preparedness of medical school graduates for the internship programme is one of the most important objectives of undergraduate training. The experience of internship often determines the future course of a doctor’s career and fosters feelings of preparedness for practice. Psychiatry training has been ranked as contributing the least to feeling prepared.^12^

In Australia, general practitioners perceived their undergraduate training to lack content on counselling skills and treatment of anxiety, depression, substance abuse, personality disorders and psychopharmacology.^13^

One in four people may suffer from mental and behavioural disorders at some point during their life, and a common perception is that only psychiatric specialists should treat these patients. The reality is that the number of psychiatrists and psychiatric facilities is woefully inadequate.^14^ To bridge the mental healthcare gap, mental healthcare is included in undergraduate curricula to enable primary care physicians to treat patients with mental disorders and to acquire the knowledge, skills and confidence to manage patients with mental illnesses.^15^

Even beyond primary psychiatric care, existing literature suggests that better undergraduate medical training in psychiatry can serve to improve the knowledge of psychiatric conditions among general medical practitioners, and may be the most important modifiable influence on recruitment into psychiatry.^16,17^

Medical students from the University of Pretoria are exposed to mentally ill patients during their clinical rotations in psychiatry. It is here where they are expected to learn about various psychiatric disorders and gain knowledge about interviewing and treating patients with mental illnesses. It is therefore important to know if they are adequately equipped with the knowledge and skills to deal with issues relating to mental health, as would arise in primary healthcare.

This study aimed to evaluate medical students’ perceptions of their career readiness as future doctors after their clinical rotation in psychiatry.

## Methods

### Study design

This was a retrospective, cross-sectional, quantitative analytical study.

### Objectives

The objectives of this study were to determine:

- The proportion of medical students who feel adequately prepared for their role as medical practitioners.
- The medical students’ perceptions of their exposure to various psychiatric conditions.
- If there is an association between career preparedness and exposure to certain psychiatric conditions.
- If associated factors, including exposure to mentally ill patients, interviewing skills and knowledge about prescribing appropriate psychiatric medications, influence medical students’ perceptions of career preparedness.

### Research setting

This study took place at the University of Pretoria, South Africa. The medical undergraduate curriculum is a 6-year course, including 54 months of theoretical training and thereafter an 18-month clinical rotation, called the Student Intern the Student Intern Complex (SIC). Medical students in this 18-month rotation are often referred to as student interns or ‘SICs’ and are considered to be in their final ‘year’ of medical school. They graduate after successful completion of this undergraduate training, and are then required to work as medical interns (junior doctors) during their 2-year internship.

During the Student Intern Complex, the medical students rotate through the various clinical disciplines at 7-week intervals. These rotations take place at training hospitals that are affiliated to the University of Pretoria.

The 7-week clinical rotation in psychiatry takes place at two hospitals. The medical students rotate at Weskoppies Hospital, which is a specialist psychiatric hospital, and at Steve Biko Academic Hospital, which is a tertiary hospital with a specialised psychiatric unit.

During the rotation in psychiatry, students receive theoretical and clinical training. Theory is taught through student-led presentations as well as associated small-group training by psychiatrists. The emphasis is on clinical teaching. They are exposed to both inpatients and outpatients with mental illnesses. Medical students have contact with inpatients during ward rounds, under the supervision of psychiatry registrars, as well as consultant-led grand rounds. They also gain experience by learning to take an appropriate history and perform mental state examinations when interviewing mentally ill patients who are to be admitted to either of the hospitals. At the outpatient department or clinic, the medical students interview mentally ill patients and then present their findings to a psychiatry registrar. It is here where they practise the appropriate prescribing of medication.

Following their rotation in psychiatry, medical students are asked to complete a questionnaire about their experience during the rotation. This questionnaire was designed to gather feedback from the medical students regarding their experience of the rotation, with the aim of improving future teaching during the clinical rotation. This questionnaire is completed voluntarily and anonymously.

### Population and sample

The study included medical students during their clinical rotation in psychiatry at Weskoppies Hospital and Steve Biko Academic Hospital. The group of medical students rotating at these hospitals from 2011 to 2015 was selected. During this period, data from 770 completed questionnaires were available and included in the study.

### Instrument and procedure

The questionnaire was not created for the specific purpose of this study, but was compiled by the Department of Psychiatry and has been used to evaluate student experiences of the rotation since 2001. The main purpose of these questionnaires is to identify components of the curriculum where improvements could be made. In 2015, Du Preez et al. used the same questionnaire to assess learning opportunities and the quality of learning experienced by students at the University of Pretoria.^15^

The questionnaire consists of three sections. Section A includes closed questions (Yes/No/Don’t know) on exposure to patients with various psychiatric diagnoses, ethical considerations and assessment of patients under the Mental Health Care Act.^18^ Section B uses Likert scales (1–5) to evaluate the student interns’ perceptions on the experience of the rotation. On the Likert scale, 5 represents ‘strongly agree’ and 1 represents ‘strongly disagree’. Section C of the questionnaire includes open-ended questions regarding the strengths and weaknesses of the rotation and possible areas of improvement.

The study was carried out retrospectively, using data from these previously completed questionnaires. Questionnaires from a 5-year period were used, that is, 2011–2015 (*N* = 770).

To address the objectives of this study, only relevant questions were selected. All the questions from Section A relating to exposure to various psychiatric conditions were included. These conditions were post-traumatic stress disorder (PTSD), panic disorder, social anxiety disorder, obsessive-compulsive disorder (OCD), adjustment disorders, bipolar disorders, schizophrenia, other psychotic disorders (these include brief psychotic disorder, delusional disorder, schizophreniform disorder, schizoaffective disorder, substance/medication-induced psychotic disorder and psychotic disorder due to another medical condition), personality disorders, alcohol use disorder and other substance use disorders. Four questions from Section B were used; these included questions relating to the medical students’ perceptions about:

- the usefulness of the psychiatry rotation in their preparation as a doctor
- their exposure to mentally ill patients
- their familiarity with interviewing a patient with mental illness
- their familiarity with prescribing psychiatric medications.

None of the open-ended questions from Section C of the questionnaire were used in the study.

### Data analysis

Descriptive statistics relating to the objectives were summarised in frequency tables and graphically represented, after which the contribution to students’ perceptions of usefulness was estimated. Likert scale outcomes were then binarised as useful (4,5) or not useful (1,2,3). The proportion of medical students who found the rotation in psychiatry useful was estimated along with a 95% confidence interval, both overall and by year.

The medical students’ exposure to different conditions was determined and a Pearson’s Chi-square test was employed to compare years with respect to exposure proportions. Those who marked ‘I don’t know’, with regard to exposure to a certain psychiatric condition, were excluded from the analysis.

To determine the association between the medical students’ perception of the usefulness of the rotation and their exposure to different conditions, crude and adjusted odds ratios (OR) were determined along with 95% confidence intervals. Odds ratios were followed from logistic regression analyses. This was done overall and by year.

Logistic regression analyses were also used to determine if associated factors influenced the students’ perception of preparedness. Crude and adjusted OR (per year) were determined along with 95% confidence intervals. The associated factors were the medical students’:

- perception of sufficient exposure to mentally ill patients
- familiarity with interviewing skills
- familiarity with prescribing psychiatric medications.

### Ethical considerations

This study was approved by the Research Ethics Committee, Faculty of Health Sciences at the University of Pretoria (Ethics Reference No. 370/2016). Approval to make use of the questionnaires completed by medical students was obtained from the Executive Committee of the School of Medicine of the University of Pretoria. Participant confidentiality was ensured as the questionnaire was completed anonymously. Informed consent from medical students was implied as the questionnaire states that completion of the questionnaire acknowledges informed consent for evaluation and research purposes. The questionnaire also states that the medical students’ participation is voluntary.

## Results

### Medical students’ perceptions of usefulness of the psychiatry rotation

Table 1 shows the proportion of medical students who felt adequately prepared for their role as medical practitioners, both overall and by year. Overall, 93.10% of medical students felt adequately prepared for their role as medical practitioners after their clinical rotation in psychiatry. Based on the Pearson’s Chi-square test, years were found not to differ significantly (*p* = 0.151) with respect to the proportion of students who felt adequately prepared for their role as medical practitioners.

**TABLE 1:** Proportion of medical students who feel adequately prepared for their role as medical practitioners, overall and by year.

Year	*N*	*n*	Preparedness (%)	95% confidence interval
Overall	754	702	93.10	(91.05; 94.81)
2011	169	158	93.49	(88.65; 96.71)
2012	177	170	96.05	(92.02; 98.40)
2013	207	187	90.34	(85.47; 94.00)
2014	124	113	91.13	(84.68; 95.49)
2015	77	74	96.10	(89.03; 99.19)

*N* = Number of students who answered that question.

*n* = Number of students who agreed that the rotation was useful in their preparation as a doctor.

### Exposure to various psychiatric disorders

**FIGURE 1:**
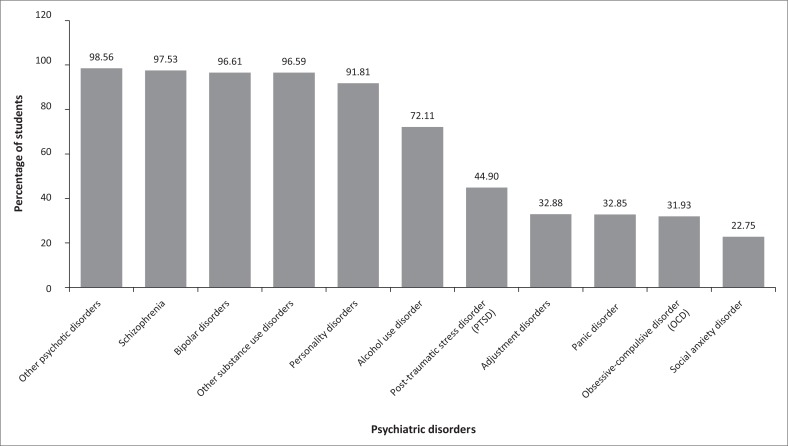
Proportion of students (%) exposed to different disorders from 2011 to 2015.

Figure 1 shows the proportion of students exposed to different psychiatric disorders from 2011 to 2015. Medical students were most frequently exposed to mentally ill patients with other psychotic disorders (98.56%), schizophrenia (97.53%), bipolar disorders (96.61%), substance use disorders, other than alcohol use disorder, (96.59%) and personality disorders (91.81%). Fewer students were exposed to mentally ill patients with PTSD (44.90%), adjustment disorder (32.88%), panic disorder (32.85%), OCD (31.93%) and social anxiety disorder (22.75%).

**FIGURE 2:**
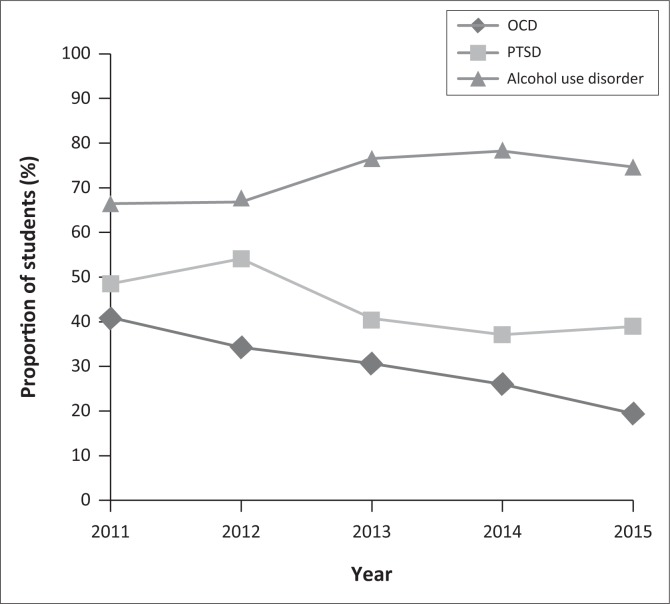
Proportion of students exposed to post-traumatic stress disorder (PTSD), obsessive-compulsive disorder (OCD) and alcohol use disorder by year (%).

The proportion of students exposed to various psychiatric conditions, by year, is shown in Table 2. The proportion of students exposed to OCD, PTSD and alcohol use disorder was found to vary significantly across years, as shown in Figure 2.

**TABLE 2:** Differences between year groups with respect to proportion of students exposed to various psychiatric conditions.

Mental disorders	Proportion of students exposed % (95% confidence interval)						*p*-value
	*n* = number of students exposed to psychiatric condition						
	2011–2015	2011	2012	2013	2014	2015	
OCD	**31.93** (28.63; 35.38 )	**40.94** (33.49; 48.70)	**34.25** (27.37; 41.66)	**30.62** (24.45; 37.35)	**26.02** (18.52; 34.70)	**19.48** (11.33; 30.09)	0.006*
	*n* =243	*n* = 70	*n* = 62	*n* = 64	*n* = 32	*n* = 15	
PTSD	**44.90** (41.35; 48.50 )	**48.54** (40.84; 56.29)	**54.14** (46.59; 61.56)	**40.76** (34.06; 47.72)	**37.10** (28.60; 46.23)	**38.96** (28.05; 50.75)	0.012**
	*n* = 343	*n* = 83	*n* = 98	*n* = 86	*n* = 46	*n* = 30	
Alcohol use disorder	**72.11** (68.76; 75.29 )	**66.47** (58.76; 73.58)	**66.85** (59.42; 73.72)	**76.56** (70.22; 82.12)	**78.23** (69.92; 85.13)	**74.67** (63.30; 84.01)	0.046**
	*n* = 543	*n* = 111	*n* = 119	*n* = 160	*n* = 97	*n* = 56	
Bipolar disorder	**96.61** (95.07; 97.77 )	**97.66** (94.12; 99.36)	**95.05** (90.82; 97.71)	**98.10** (95.20; 99.48)	**93.65** (87.87; 97.22)	**98.70** (92.98; 99.97)	0.102
	*n* = 740	*n* = 167	*n* = 173	*n* = 206	*n* = 118	*n* = 76	
Adjustment disorder	**32.88** (29.51; 36.39 )	**32.10** (24.99; 39.88)	**30.34** (23.68; 37.66)	**39.23** (32.57; 46.21)	**30.77** (22.57; 39.97)	**26.32** (16.87; 37.68)	0.191
	*n* = 244	*n* = 52	*n* = 54	*n* = 82	*n* = 36	*n* = 20	
Schizophrenia	**97.53** (96.17; 98.51 )	**98.27** (95.02; 99.64)	**98.36** (95.28; 99.66)	**95.73** (92.06; 98.03)	**96.83** (92.07; 99.13)	**100.00** (95.32; 1.00)	0.203
	*n* = 751	*n* = 170	*n* = 180	*n* = 202	*n* = 122	*n* = 77	
Other substance use disorders	**96.59** (95.04; 97.76 )	**95.93** (91.79; 98.35)	**98.34** (95.23; 99.66)	**95.19** (91.34; 97.67)	**98.39** (94.30; 99.80)	**94.81** (87.23; 98.57)	0.277
	*n* = 736	*n* = 165	*n* = 178	*n* = 198	*n* = 122	*n* = 73	
Personality disorders	**91.81** (89.62; 93.66 )	**90.59** (85.17; 94.52)	**94.44** (90.02; 97.30)	**90.38** (85.54; 94.03)	**92.74** (86.67; 96.63)	**90.67** (81.71; 96.16)	0.586
	*n* = 695	*n* = 154	*n* = 170	*n* = 188	*n* = 115	*n* = 68	
Other psychotic disorders	**98.56** (97.44; 99.28 )	**98.24** (94.93; 99.63)	**98.91** (96.11; 99.87)	**98.09** (95.17; 99.48)	**98.40** (94.34; 99.81)	**100.00** (95.26; 1.00)	0.783
	*n* = 752	*n* = 167	*n* = 181	*n* = 205	*n* = 123	*n* = 76	
Panic disorder	**32.85** (29.51; 36.32 )	**35.88** (28.68; 43.58)	**32.04** (25.32; 39.37)	**30.48** (24.33; 37.18)	**35.00** (26.52; 44.24)	**31.17** (21.09; 42.74)	0.800
	*n* = 249	*n* = 61	*n* = 58	*n* = 64	*n* = 42	*n* = 24	
Social anxiety disorder	**22.75** (19.81; 25.91 )	**20.93** (15.11; 27.78)	**23.33** (17.36; 30.20)	**24.52** (18.83; 30.95)	**22.31** (15.25; 30.78)	**21.33** (12.71; 32.32)	0.935
	*n* = 172	*n* = 36	*n* = 42	*n* = 51	*n* = 27	*n* = 16	

* , Highly significant.

** , Statistically significant.

OCD, obsessive-compulsive disorder; PTSD, post-traumatic stress disorder.

Based on Pearson’s chi-square test, years were found to differ significantly with respect to the proportion of students exposed to OCD (*p* = 0.006), with a much lower exposure noted after 2013. During 2011–2013, 34.94% of students were exposed to patients with OCD, compared to 23.50% during 2014–2015. This difference is statistically significant (*p* = 0.003).

With respect to the proportion of students exposed to PTSD, years were also found to differ significantly (*p* = 0.012). In particular, a higher exposure rate was observed before 2013, compared to 2013 onwards. During 2011–2013, 47.42% of students were exposed to PTSD, compared to 37.81% during 2014–2015. This difference is also statistically significant (*p* = 0.019).

The proportion of students exposed to alcohol use disorders was also found to differ significantly over the years (*p* = 0.012). An especially high exposure was noted from 2013 onwards. From 2011 to 2012, 66.67% of students were exposed to patients with alcohol use disorder. This is significantly less (*p* = 0.002) than 76.72% of students who were exposed to these patients from 2014 to 2015.

Differences between the years, with regard to proportions of students exposed to other psychiatric conditions, were not statistically significant (Table 2).

### Medical student’s perception of preparedness and their exposure to various psychiatric conditions

There is no statistically significant association between career preparedness and exposure to any specific psychiatric condition, as is demonstrated in Table 3. Students who were exposed to patients with schizophrenia were 2.63 times more likely to feel prepared for their career than those students who were not (*p* = 0.221).

**TABLE 3:** The association between medical students’ perception of preparedness and their exposure to various psychiatric conditions, as an odds ratio for preparedness.

Psychiatric condition	Crude odds ratio (95% confidence interval)	*p*	Adjusted odds ratio* (95% confidence interval)	p*
Schizophrenia	2.63 (0.74; 9.32)	0.135	2.23 (0.62; 8.00)	0.221
Bipolar disorder	1.88 (0.54; 6.51)	0.318	1.93 (0.55; 6.81)	0.306
OCD	1.62 (0.84; 3.16)	0.153	1.62 (0.83; 3.17)	0.160
Other psychotic disorders	1.49 (0.19; 12.03)	0.706	1.32 (0.16; 10.76)	0.794
PTSD	1.22 (0.69; 2.16)	0.502	1.15 (0.64; 2.05)	0.636
Adjustment disorder	1.17 (0.63; 2.18)	0.625	1.25 (0.67; 2.35)	0.480
Panic disorder	1.10 (0.60; 2.03)	0.752	1.10 (0.60; 2.03)	0.759
Alcohol disorder	0.98 (0.52; 1.84)	0.939	1.05 (0.55; 1.99)	0.891
Social Anxiety disorder	0.70 (0.37; 1.32)	0.270	0.71 (0.38; 1.34)	0.290
Personality disorders	0.44 (0.11; 1.87)	0.269	0.42 (0.10; 1.79)	0.243

* , Adjusted for year.

OCD, obsessive-compulsive disorder; PTSD, post-traumatic stress disorder.

Schizophrenia is therefore the psychiatric condition that is associated with the highest perception of career preparedness. Interestingly, students were only 0.44 times more likely to feel prepared to work as a general practitioner after exposure to patients with personality disorders (*p* = 0.269). Crude OR and those adjusted for year of study did not differ considerably. Further adjusted OR are shown in Table 3.

The OR for students exposed to mentally ill patients with substance use disorders could not be estimated overall, as all of the students who reported not being adequately prepared for their careers were exposed to patients with substance use disorders. Of the students who felt prepared, 96.26% of students were exposed to mentally ill patients with alcohol use disorder.

### Associated factors affecting medical students’ perception of preparedness

Most of the medical students (95.23%, 95% confidence interval [CI] 93.45; 96.63) felt comfortable with interviewing patients with a mental illness. Many students (74.74%, 95% CI 71.49; 77.79) were of the opinion that their exposure to patients with a mental illness was sufficient and 65.79 (95% CI 62.28; 69.16) were comfortable with prescribing psychiatric medication.

Table 4 shows crude OR (2011–2015) and adjusted OR (for years over the study period) for other factors that may influence medical students’ perceptions of career preparedness. When adjusted for year of study, OR were higher. Both overall and by year, the results were found to be statistically significant.

**TABLE 4:** Crude and adjusted odds ratios for other factors associated with students’ perception of preparedness for their careers.

Factor	Crude odds ratio	95% confidence interval	*p*-value	Adjusted odds ratio	95% confidence interval	*p*
Interviewing skills	15.04	(6.25; 36.19)	<0.001	17.16	(6.75; 43.64)	<0.001
Prescribing medications	4.90	(2.21; 10.90)	<0.001	5.58	(2.47; 12.61)	<0.001
Sufficient exposure to patients	5.49	(2.68; 11.24)	<0.001	5.51	(2.63; 11.52)	<0.001

Students who were comfortable with interviewing mentally ill patients were 17.16 times more likely to feel prepared for their future careers (*p* = 0.001). Sufficient exposure to mentally ill patients and knowledge of prescribing medication were also important in affecting their perception of career preparedness (OR 5.51 and OR 5.58, respectively). Both these findings were statistically significant (*p* < 0.001).

## Discussion

The purpose of undergraduate training in medical school is to lay the educational foundations for a lifelong career and to prepare junior doctors for the first stage in their working lives.^19^ Doctors have often complained that little attention is given during undergraduate training about what to expect in the working environment.^20^

The proportion of medical students in this study (93.10%) who felt adequately prepared for their future careers as doctors was higher compared to international studies.^3^ For example, in a survey of newly qualified doctors in the United Kingdom, only 4.3% strongly agreed, and 32% agreed, that their training had prepared them well for the work they had performed.^19^ The results in this study differ significantly from those reported internationally.

Repeating a study such as this, after medical students have worked as junior doctors in their internship, may produce different results. These junior doctors will have a better idea of the skills and knowledge required.

Undergraduate psychiatric training should be relevant and useful to all future doctors. Medical students have to be able to recognise and treat common psychiatric illnesses that they may encounter in general practice or other disciplines of medicine. These disorders may include disorders not usually treated in specialised psychiatric facilities and less commonly encountered disorders such as PTSD, adjustment disorders, panic disorder, OCD and social anxiety disorder. A previous study highlighting learning priorities of medical students agreed that basic psychiatric skills needed by most doctors were more important than specialised psychiatric knowledge.^20^

In this study, the medical students were mostly exposed to conditions considered to be severe mental illnesses, such as schizophrenia, other psychotic disorders and bipolar disorders. These conditions are considered severe because they negatively impact a patient’s level of functioning and often require inpatient psychiatric treatment. In addition, inpatient admissions for patients with severe mental illness tend to be prolonged, thus increasing medical students’ exposure to these conditions. In this study, a large proportion of students were exposed to substance use disorders as well as personality disorders. This finding is in keeping with the high rate of comorbid substance abuse in patients with severe mental illness. The rates of comorbidity with substance-related disorders are high in schizophrenia, and substance use disorders occur in over half of individuals with bipolar 1 disorder. Furthermore, there is a high rate of co-occurrence between certain personality disorders and specific psychiatric illnesses.^21^

Significantly fewer medical students were exposed to patients with OCD and PTSD after 2013, which may reflect a change in the profile of mentally ill patients admitted to hospitals for inpatient care. Since 2011, the availability of beds at Weskoppies Hospital decreased, thus necessitating the prioritisation of beds for patients who are more severely ill, with a higher risk of injury to self or others. Fewer beds are therefore available for patients with mental illnesses that have little or no behavioural problems such as OCD and PTSD. The same is true for panic disorder, adjustment disorder and social anxiety disorder.

At Weskoppies Hospital, patients who have a mental illness with co-morbid substance use disorders are referred to the substance rehabilitation unit (SRU). This is a 6-week, inpatient treatment programme for dependency on psychoactive substances. The SRU was established during 2012, and may account for more students being exposed to alcohol use disorder from 2013 onwards.

In this study, students’ perceptions of the usefulness of the psychiatric rotation were not influenced by exposure to any one psychiatric condition. Students in their final stages of training are often more focused on attaining skills they may require for their work after medical school. Oakley reported that students place more emphasis on skills such as assessment of suicide risk and self-harm, management of alcohol withdrawal and recognition of delirium.^20^ These skills were ranked above skills that are more specific to psychiatrists, such as the management of bipolar disorders and schizophrenia.^20^ Our study shows contrasting results in that medical students were more likely to feel prepared after exposure to patients with schizophrenia and bipolar disorders than to patients with, for example, personality and anxiety disorders. These findings may be more representative of the mentally ill patient profile at tertiary hospitals in the country, and that junior doctors working at district level would be expected to manage acute cases of psychosis and mood episodes associated with severe mental illnesses such as schizophrenia and bipolar disorders.

The students placed a lot of emphasis on the importance of confidently being able to interview a patient with a psychiatric illness. Sufficient exposure to mentally ill patients, as well as knowledge of prescribing appropriate psychiatric medications to treat patients with mental illnesses, was also noted to contribute significantly to the overall feeling of preparedness. Previous studies have noted concerns that undergraduate education does not adequately prepare doctors for their early working lives. One study showed that junior doctors and their consultants felt that they were best prepared in areas of communication skills but less prepared in basic clinical competences such as treatment, prescribing and managing emergencies.^19^ Our study shows that clinical exposure and ‘hands-on’ experience when managing patients with mental illnesses is an effective way to teach psychiatry at an undergraduate level.

The majority of students considered themselves to be career ready after their final psychiatry rotation, with no specific clinical exposure influencing this outcome. Medical students at the University of Pretoria rotate through many other clinical disciplines during their final 18 months as medical students. The knowledge and experience gained while rotating through these disciplines may also contribute to their overall feeling of preparedness. In addition to this, the majority of the clinical exposure during the rotation was to severe mental illnesses, which is not representative of the psychiatric pathology seen in general practice. This suggests that medical students may not be fully aware of the competencies required for general practice. Furthermore, previous studies have shown that self-perceived competence does not correlate well with objectively measured competence in medical students.^22^

The results of our study are limited by the cross-sectional design. Additionally, the Diagnostic and statistical manual of mental disorders, fifth edition (DSM-5), was implemented in 2013, during the study period. Despite there being a transitional period where both the DSM-IV and DSM-5 were used concurrently, the questionnaire used in the study was not updated with DSM-5 terminology.

Fortunately, most disorders were listed individually and not grouped under broader categories; for example, OCD and PTSD were listed specifically and not named as being ‘anxiety disorders’ in the questionnaire. Furthermore, although not specifically designed for the purpose of this study, the questionnaire used does not address certain mood disorders commonly encountered in both general and specialist practice, including major depressive disorder and persistent depressive disorder. This omission is seen as a significant limitation of the study.

Students’ opinions on their ability to diagnose and treat depressive disorders would have contributed greatly to this study as these disorders are commonly encountered in general practice. Aside from including more of the common mental disorders in the questionnaire, we suggest adding questions that assess whether students feel prepared to manage specific scenarios they will commonly encounter in their initial years of being a doctor. These include questions relating to managing substance withdrawal, aggression, assessing suicide risk and recognising delirium.

In summary, these results demonstrate that the present clinical rotation for training in psychiatry has value. They also emphasise the importance of teaching aspects of mental illness that will be encountered in everyday general medical practice. Integrating psychiatry teaching into other disciplines of medicine may be of value in this regard.

## Conclusion

It appears that the undergraduate curriculum for training in psychiatry, especially the clinical rotation, has value in preparing medical students for their internship. This is evident from the results of this study, which indicate that the overwhelming majority of medical students at the University of Pretoria feel prepared for their role as doctors. More exposure to certain disorders that general practitioners will encounter in practice will potentially prepare students better for their careers. These include disorders such as PTSD, OCD and panic disorder, among others. There is no evidence that exposure to any specific psychiatric condition is associated with better career preparedness. Overall, clinical training seems to be an effective way to teach psychiatry to undergraduate medical students as interviewing skills, knowledge about the appropriate prescribing of medications and sufficient exposure to mentally ill patients significantly affect medical students’ perceptions of career preparedness.
