# Corrigendum: Do medical students feel career ready after their psychiatry clinical rotation?

**DOI:** 10.4102/sajpsychiatry.v27i0.1773

**Published:** 2021-11-08

**Authors:** Kim Ives, Piet J. Becker, Gian Lippi, Christa Krüger

**Affiliations:** 1 Department of Psychiatry, Faculty of Health Sciences, University of Pretoria, Pretoria, South Africa; 2 Research Office, Faculty of Health Sciences, University of Pretoria, Pretoria, South Africa

In the version of the article initially published, Ives K, Becker PJ, Lippi G, Krüger C. Do medical students feel career ready after their psychiatry clinical rotation?. S Afr J Psychiat. 2019;25(0), a1397. https://doi.org/10.4102/sajpsychiatry.v25i0.1397, the name of the last author was given incorrectly. The last author’s name should be listed as Christa Krüger instead of Christina Krüger. This was updated in the ‘Authors’ section.

This correction does not alter the study’s findings of significance or overall interpretation of the study’s results. The authors apologise for any inconvenience caused.

